# Supplementary material for: Combined Use of Fatty Acid Profiles and Elemental Fingerprints to Trace the Geographic Origin of Live Baits for Sports Fishing: The Solitary Tube Worm (Diopatra neapolitana, Annelida, Onuphidae) as a Case Study
Source: Animals (Basel). 2024 Apr 30;14(9):1361. doi: 10.3390/ani14091361 (PMC11083138; doi:10.3390/ani14091361)
Supplement: Supplementary file 1 [file animals-14-01361-s001.zip › animals-2896763-supplementary.pdf]

Supplementary material

Article

Combined use of fatty acid profiles and elemental fingerprints to trace the geographic origin of live baits for sports fishing: the solitary tube worm (*Diopatra neapolitana*) as a case study

Fernando Ricardo [1*], Marta Lobão Lopes [1], Renato Mamede [1], M. Rosário Domingues [2,3], Eduardo Ferreira da Silva [4], Carla Patinha [4] and Ricardo Calado [1*]

Figure S1. Ratios of elements to Calcium (Ca) concentrations (mg to mg) (average ±SD; n=10) of *Diopatra neapolitana* jaws from four locations within the coastal lagoon Ria de Aveiro, mainland Portugal: Espinheiro Channel (E:), Ílhavo Channel (I) and Mira Channel (M1 and M2). Different letters indicate significant differences among sampling locations at $p < 0.05$.

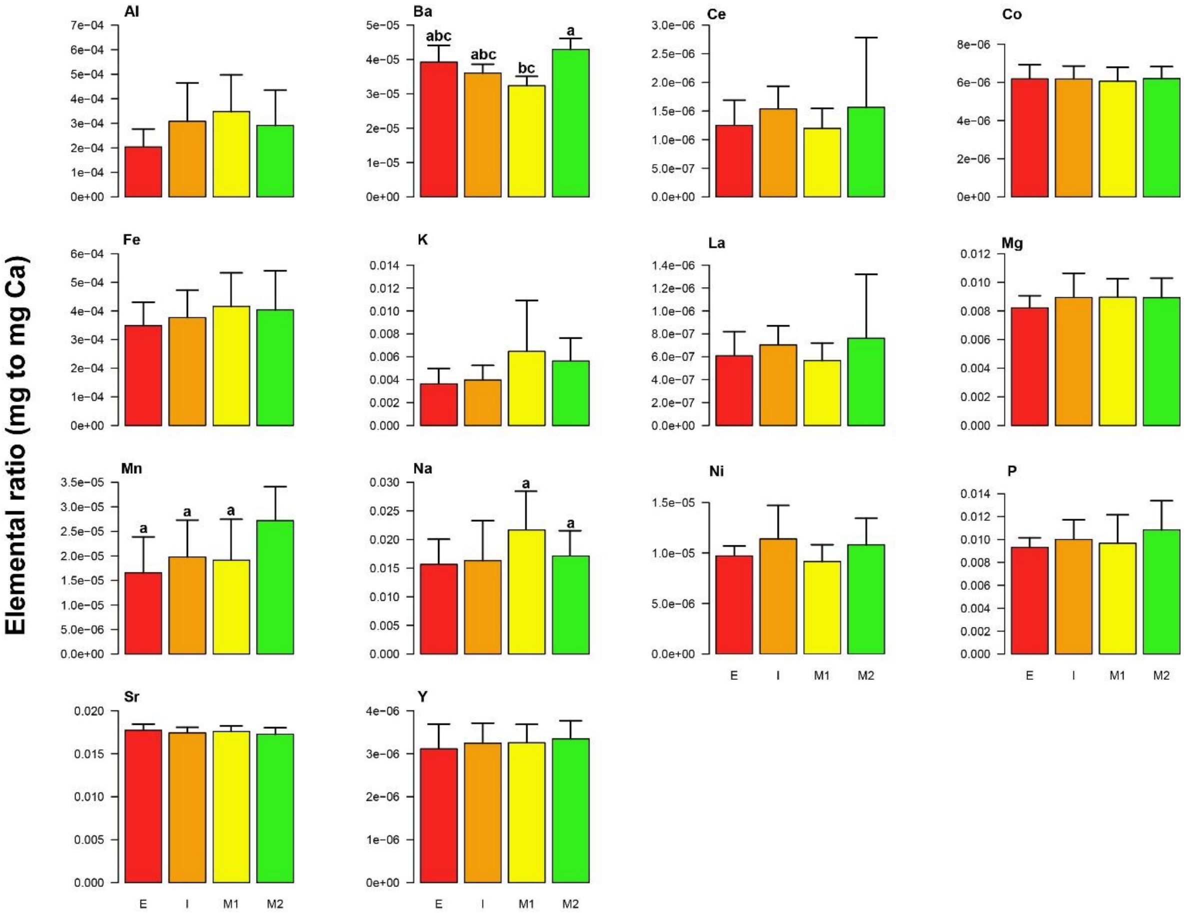

Table S1 – Fatty acid profiles (data presented as percentage of relative abundances) of the whole body of *Diopatra neapolitana* (values are means of 10 replicates±SD) from Espinheiro (E), Ílhavo (I) and Mira (M1 and M2) channels in the coastal lagoon Ria de Aveiro, mainland Portugal. SFAs – saturated fatty acids; MUFAs – monounsaturated fatty acids; and PUFAs – polyunsaturated fatty acids. Different superscript letters indicate significant differences among sampling locations at $p < 0.05$.

Fatty Acid (%)	Espinheiro (E)	Ilhavo (I)	Mira 1 (M1)	Mira 2 (M2)
14:0	3.49 ± 1.18	2.34 ± 0.75	2.72 ± 1.06	1.82 ± 0.70
15:0	1.22 ± 0.32[a]	0.96 ± 0.31	0.79 ± 0.25	0.85 ± 0.30
16:0	24.41 ± 4.79[a]	22.82 ± 7.59	21.33 ± 6.56	18.49 ± 5.06
17:0	2.48 ± 0.35[a]	2.54 ± 1.02	2.03 ± 0.97	2.14 ± 0.41
18:0	12.53 ± 1.62	17.43 ± 3.90[a]	17.31 ± 5.09[a]	12.91 ± 1.58
20:0	1.21 ± 0.39	1.38 ± 0.53	1.84 ± 0.55	1.08 ± 0.42
21:0	0.52 ± 0.14	0.57 ± 0.20	0.49 ± 0.13	0.51 ± 0.20
22:0	2.20 ± 0.85	2.67 ± 1.02	2.34 ± 0.93	1.97 ± 0.69
**∑SFA**	**48.06 ± 9.64**	**50.71 ± 15.32**	**48.85 ± 15.53**	**39.77 ± 9.36**
16:1*n*-7	2.11 ± 0.75	1.19 ± 0.46	1.41 ± 0.41[a]	2.14 ± 0.62
18:1*n*-9	2.40 ± 0.74	1.66 ± 0.37	1.84 ± 0.82	1.60 ± 0.35
18:1*n*-7	7.57 ± 1.89[a]	5.32 ± 1.51	2.94 ± 1.42	6.63 ± 1.93
20:1*n*-9	4.36 ± 0.42	4.61 ± 0.49	4.07 ± 0.44	3.81 ± 0.30
20:1*n*-7	2.52 ± 0.34	2.69 ± 0.54	2.66 ± 0.34	2.23 ± 0.41
22:1*n*-11	0.68 ± 0.12	0.83 ± 0.12	0.58 ± 0.22	0.70 ± 0.11
**∑MUFA**	**19.64 ± 4.25**	**16.32 ± 3.51**	**13.50 ± 3.64**	**17.11 ± 3.72**
18:2*n*-6	0.94 ± 0.26[a]	0.46 ± 0.21	0.63 ± 0.29	0.98 ± 0.22
18:3*n*-3	2.61 ± 1.04	2.21 ± 1.16	1.28 ± 0.56	2.28 ± 0.58
20:2*n*-9	2.97 ± 0.44	2.64 ± 0.43	2.17 ± 0.47	2.48 ± 0.35
20:3*n*-3	1.19 ± 0.44	1.40 ± 0.38	0.98 ± 0.38	1.02 ± 0.27
20:4*n*-6	1.87 ± 0.70	2.15 ± 1.08	2.00 ± 1.15[a]	5.21 ± 2.09
20:5*n*-3	7.59 ± 3.58	6.41 ± 2.58	11.78 ± 4.97	10.95 ± 3.47
22:1*n*-11	0.01±0.00	0.01±0.00	0.01±0.00	0.01±0.00
22:2*n*-9	7.24 ± 1.45	8.93 ± 1.56	8.38 ± 1.24	7.53 ± 1.45
22:3*n*-6	1.46 ± 0.37	1.17 ± 0.28	0.93 ± 0.38	1.04 ± 0.35
22:4*n*-6	2.65 ± 1.06	2.68 ± 0.90	3.78 ± 1.47[a]	5.99 ± 1.94
22:6*n*-3	3.77 ± 1.39	4.91 ± 1.83	5.80 ± 2.31	5.64 ± 1.81
**∑PUFA**	**32.3 ± 10.73**	**32.96 ± 10.41**	**37.83 ± 13.22**	**43.12 ± 12.53**

Table S2 – Permutational multivariate analysis of variance (PERMANOVA) among fatty acid profiles of whole body, elemental ratios of jaws and combination of fatty acid profiles of whole body and elemental ratios of jaws of the polychaete *Diopatra neapolitana* sourced from Espinheiro Channel (E:), Ílhavo Channel (I) and Mira Channel (M1 and M2). Significant differences $p < 0.05$.

Structure (s) Fingerprint (s)	Location	E	I	M1
Body - Fatty acid profile	I	<0.001	-	-
	M1	<0.001	0.013	-
	M2	<0.001	0.007	0.356
Jaws - Elemental fingerprint	I	0.333	-	-
	M1	0.024	0.109	-
	M2	0.009	0.009	<0.001
Body & Jaws - Fatty acid profile & Elemental fingerprint	I	<0.001	-	-
	M1	<0.001	0.029	-
	M2	<0.001	0.001	0.008
